# A longitudinal serum NMR-based metabolomics dataset of ischemia-reperfusion injury in adult cardiac surgery

**DOI:** 10.1038/s41597-020-0545-0

**Published:** 2020-06-24

**Authors:** Raluca Georgiana Maltesen, Reinhard Wimmer, Bodil Steen Rasmussen

**Affiliations:** 1 Department of Anaesthesia and Intensive Care, Aalborg University Hospital, 9000 Aalborg, Denmark; 2 Department of Chemistry and Bioscience, Aalborg University, 9220 Aalborg, Denmark; 3 Department of Clinical Medicine, Faculty of Medicine, Aalborg University, 9220 Aalborg, Denmark

**Keywords:** Trauma, Outcomes research, Time series, Metabolomics, NMR spectroscopy

## Abstract

Cardiovascular disease is the leading cause of death worldwide and cardiac surgery is a key treatment. This study explores metabolite changes as a consequence of ischemia-reperfusion due to cardiac surgery with the use of cardiopulmonary bypass (CPB). To describe the ischemia-reperfusion injury, metabolite changes were monitored in fifty patients before and after CPB at multiple time points. We describe a longitudinal metabolite dataset containing nearly 600 serum nuclear magnetic resonance (NMR) spectra obtained from samples collected simultaneously from the pulmonary artery (deoxygenated blood) and left atrium (oxygenated blood) before ischemia (pre-CPB), immediately after reperfusion (end-CPB), and the following 2, 4, 8, and 20 hours postoperatively. In addition, a longitudinal dataset including 57 quantified metabolites is also provided. These datasets will help researchers studying ischemia-reperfusion injury, as well as the time-dependent alterations related to the surgical trauma and the subsequent processes required in regaining metabolite balance. The datasets could also be used for the development of processing algorithms for NMR-based metabolomics studies and methods for the analysis of longitudinal multivariate data.

## Abstract (summary)

**Table:** 

Measurement(s)	human blood serum metabolite
Technology Type(s)	one-dimensional nuclear magnetic resonance spectroscopy
Factor Type(s)	cardiac surgery • time series • pulmonary artery and left atrium
Sample Characteristic - Organism	Homo sapiens

Machine-accessible metadata file describing the reported data: 10.6084/m9.figshare.12249065

## Background & Summary

Cardiovascular disease is the leading cause of death in many countries^1^. The vast majority of cardiac patients suffer from coronary artery disease, which is characterized by the build-up of cholesterol deposits in the wall of the coronary arteries. This leads to impaired blood flow with patients suffering chest pain and heart attacks^2,3^. Part of this population need cardiac surgery. Coronary artery bypass grafting (CABG) is considered the gold standard surgical procedure^4^, with more than one million surgeries being performed annually worldwide. Eighty percent of the procedures are performed with the use of a cardiopulmonary bypass (CPB) circuit^4,5^. CPB deprives the lungs of blood flow, with the lungs being solely dependent on the minimal blood supply from the bronchial arteries. Therefore, during CPB the lungs are at risk of experiencing ischemia. After weaning from CPB, the lungs are reperfused and an ischemia-reperfusion injury can occur. The injury can further impair postoperative pulmonary function with the development of more severe complications such as acute respiratory distress syndrome^6,7^. Since the blood flow during CPB is non-pulsatile, other organs can be affected by ischemia-reperfusion. In a previous work we have shown that ischemia-reperfusion is accompanied by a series of both pulmonary and systemic changes and that the duration of CPB impacts not only the metabolic activity^8,9^, but also the fibrinolytic cascade^10^, several hours after surgery.

Shifts in the levels of lactate, citric acid cycle metabolites, purines, nicotinic acid, tyrosine, hyaluronic acid, ketones, fatty acids, and lipid metabolites were found to be associated with the surgical trauma and underlying ischemia-reperfusion^8,9^. Similar alterations were observed in another recently published work involving 90 patients randomized to receive two different pulmonary protection treatments during CPB^11^. In addition, we demonstrated that prolonged surgical stress could be related to an augmented anaerobic environment and decreased levels of fatty acids, lipids, and glycoproteins. Lastly, regaining metabolic balance after ischemia-reperfusion was found to be an energy and time demanding process, with few metabolites reaching pre-ischemic levels the first 2–4 hours postoperatively, while imbalances in several metabolite levels were still observed even after 20 hours^8,9,11^.

In this study we present the longitudinal serum metabolite dataset obtained from 50 adults undergoing cardiac surgery with the use of CPB. The results of this data set have been published^8,9^. We analysed nearly 600 serum samples collected from the pulmonary artery (deoxygenated blood) and left atrium (oxygenated blood). The samples were collected just prior to CPB, immediately after CPB (0 hours), and 2, 4, 8, and 20 hours after CPB. Samples were measured on a nuclear magnetic resonance (NMR) spectroscopy platform and metabolite changes were related to the time of sample collection and duration of CPB. The data have been deposited in the MetaboLights repository along with the relative concentration of 57 metabolites and the time on bypass for each patient^12^.

These data are one of the first longitudinal metabolite datasets enabling the investigation of the effect of ischemia-reperfusion on the human metabolome. Considering the longitudinal study design and that each patient is its own control, this clinical dataset allows for very different utilizations. Firstly, it may assist researchers studying ischemia-reperfusion models and the underlying molecular mechanisms of subsequent injury. Also, it enables the study of the time-dependent metabolite alterations required to regain balance after surgical trauma. In addition, it gives the possibility to understand the possible metabolite differences elicited by oxygenating the blood, as we simultaneously collected deoxygenated and oxygenated blood. Moreover, data can be more broadly used for the development of processing algorithms for NMR-based metabolomics studies e.g. peak picking and deconvolution, and of statistical models for the analysis of longitudinal multivariate data. Effects of data pre-treatment methods such as scaling and normalization could also be investigated with regard to metabolite ranking from the developed models or algorithms used for biological interpretation.

## Methods

**Fig. 1:**
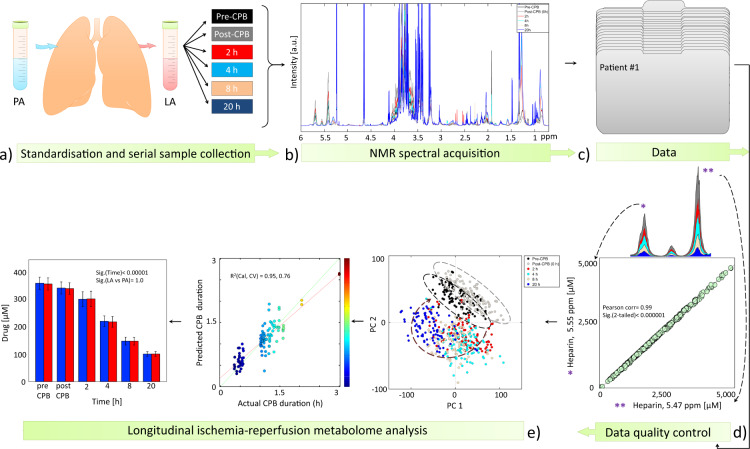
Schematic overview of the experimental and data analysis design. (**a**) A total of 50 patients were included in this study and standardized serum samples were obtained from the pulmonary artery (PA) and left atrium (LA) before cardiopulmonary bypass (Pre-CPB), at the end of CPB (Post-CPB), and at 2, 4, 8, and 20 hours postoperatively. (**b**) Samples were run on a 600 MHz nuclear magnetic resonance (NMR) spectroscopy apparat. (**c**) Processed data have been deposited at the MetaboLights database (http://www.ebi.ac.uk/metabolights) of the European Bioinformatics Institute under MTBLS88^12^. (**d**) NMR spectral data and metabolite quantification were checked by different approaches to ensure data quality. (**e**) Multivariate and univariate data analysis was performed to identify metabolite changes as a consequence of ischemia-reperfusion. Parts of the figure have been reprinted with permission^8,9^.

All procedures described here are an expansion of the previously published material and methods^8,9^. A schematic overview of the study design, sample collection, data acquisition and analysis is provided in Fig. 1.

### Patient enrolment

This study has evolved as a part of continuing efforts to understand the mechanisms involved in ischemia-reperfusion and to identify possible causes for the progression to postoperative complications such as acute lung injury. The study is registered at ClinicalTrials.gov (identifier: NCT02475694; title: Acute Lung Injury after Cardiac Surgery: Pathogenesis) and was approved by the local Ethics Committee. This prospective observational study was conducted at Aalborg University Hospital in Denmark in accordance with the Declaration of Helsinki. After obtaining informed written and oral consent, fifty consecutive patients scheduled for elective CABG with the use of CPB between September 2008 and March 2009 were included in this study. Inclusion criteria were adults above 18 years of age with hypercholesterolemia treated with statins, while exclusion criteria were patients requiring acute surgery, valve replacement, and undergoing treatment with steroids and/or other immune suppressor therapies. Demographics and selected data are provided in Table 1 and detailed in the metadata^12^. Additional information about the duration of anaesthesia, CABG, CPB, and ischemia-reperfusion are provided in the metadata.

**Table 1:** Characteristics of study population. The data are presented as numbers (n) or as means and standard deviations (SD). Abbreviation: BMI, body mass index.

Patients	n = 50
Age (years), mean ± SD	65.8 ± 9.7
Male (n)	41/50
BMI (kg/m^2^), mean ± SD	27.3 ± 4.0

### Study design

Patients fasted overnight. The anaesthesia, surgical procedure and perfusion followed the institutional standardized regimens. Immediately after anaesthesia, a pulmonary artery (PA) catheter was inserted percutaneously in the right internal jugular vein, and after sternotomy, a left atrium (LA) catheter was inserted through a surgical incision in the upper right pulmonary vein. Paired blood samples were simultaneously collected at baseline (before CPB), immediately after weaning from CPB (0 hours), and the following 2, 4, 8, and 20 hours postoperatively (Fig. 1a). Due to displacement of the catheters in four of the patients after 8 to 20 hours postoperatively, nine samples were missing from the time line. Hence, a total of 591 blood samples were collected. Serum was obtained through standard hospital protocols. To avoid variation, serum was separated at room temperature within 30 to 40 minutes from samples collection, aliquoted, and stored at −80 °C until analysis.

### NMR sample preparation and acquisition

Frozen serum samples were allowed to thaw for 30 min at 4 °C, vortexed, and subsequently centrifuged for 5 min at 12100 g and 4 °C. A total of 400 μL of the clear supernatant was mixed with 200 μL 0.20 M phosphate buffer (pH 7.4, 99% ^2^H_2_O, 0.30 mM DSA-d_6_) in a 5 mm NMR tube. During the whole process samples were kept on ice.

^1^H NMR spectra were recorded on a BRUKER AVIII-600 MHz NMR spectrometer (BrukerBioSpin, Rheinstetten, Germany) equipped with a cryogenically cooled, triple-resonance CPP-TCI probe, at a temperature of 298.1 K (25 °C). Spectral acquisition was controlled using the TopSpin 3.1 software (Bruker BioSpin, Germany). T_2_ filtered “spin-echo Carr Purcell Meiboom and Gill” (CPMG)^13^ experiments with water presaturation were acquired with the following parameters: 65536 data points over a spectral width of 20 ppm; 256 scans; 32 dummy scans; a fixed receiver gain of 203; and a relaxation delay of 4 s, during which presaturation of the water resonance was achieved by continuous irradiation at γB_1_/2π = 25 Hz. T_2_ filtering was achieved with a repeated τ-180°-τ pulse sandwich with τ = 300 μs, repeated 256 times for serum samples and 128 times for plasma samples for a total of 80 and 40 ms, respectively.

### Spectral processing

Spectral processing was carried out in TopSpin 3.1. The pre-processing steps included: artificial zero-filling by adding digital data points to the free induction decays to enhance spectral resolution; line broadening of 0.3 Hz; Fourier transformation; manual correction of spectral phase to produce pure absorption line shapes and to remove offsets in the peak shapes; correction of the baseline to zero; and calibration of the spectra to the methyl peak of L-alanine at 1.48 ppm. Spectra were reduced to buckets of 0.001 ppm width and the water region (4.65–4.95 ppm) was excluded using the AMIX software package (Analysis of MIXtures, v.3.9.10, Bruker BioSpin, Germany) (Fig. 1b). The processed metadata (Fig. 1c) is provided^12^.

### Data analysis

Data analysis was performed in MATLAB (R2011a, MathWorks) and SPSS v.22 (IBM® Statistics Inc., Armonk, NY, USA). Prior to multivariate analysis, binned data were exported to MATLAB, generalized log transformed^14^ to enhance small signals in the spectrum, normalized to the DSA-d_6_ peak intensity, and mean centred. Data quality was checked (described in details below) prior to further analysis (Fig. 1d). Unsupervised principal component analysis (PCA) was applied to find the main source of variation within the data, to check population homogeneity, and to identify outliers based on samples’ metabolic similarities and dissimilarities (Fig. 1e). Partial least square (PLS) regression analysis was applied to establish metabolome associations with the duration of ischemia-reperfusion. A ten-fold Venetian-Blind cross validation was applied for validation purposes. For PCA and PLS regression analysis the PLS-Toolbox 6.5 (Eigenvector Research, Wenatchee, WA, USA) was used. Detailed explanation for the PCA and PLS regression analysis results have been previously described^8,9^. Spectral regions contributing to sample clustering were identified and quantified. For the identification process, ^1^H shifts and their corresponding ^13^C signals were analysed by running several 2D ^1^H-^1^H total correlation spectroscopy (TOCSY) and ^1^H-^13^C heteronuclear single-quantum correlation (HSQC) spectra. Metabolite assignment was performed with the help of The Human Metabolome Database (HMDB)^15^, Bruker BBIOrefcode Database (v. 2.7.0), and literature^16,17^. A list of NMR chemical shifts found in TOCSY and HSQC spectra were reported in a previous work^18^. For the quantification process, peaks of interest were integrated using the multi-integration fitting tool implemented in the AMIX software v. 3.9.10 by using the sum of all points within a signal as the integration mode.

## Data Records

The study is registered at ClinicalTrials.gov (https://clinicaltrials.gov/ct2/show/NCT02475694). CPMG spectral data, quantified metabolites, demographic and surgical time information have been deposited at the MetaboLights database (http://www.ebi.ac.uk/metabolights) of the European Bioinformatics Institute under MTBLS881^12^.

## Technical Validation

Maintaining consistency in a sample set can be challenging, as several factors can affect the levels of metabolites. Studies have shown that pre-existing health conditions, diurnal variation, gender, age, diet, and exposure to stress factors affect the metabolome^19,20^. Hence, a number of precautions were taken into consideration during the surgical, experimental, and data analysis procedures to ensure quality and confidence in our results.

In this study patients were relatively similar with regards to their medical history (all suffering from coronary artery disease), age, body mass index, and the surgical procedure. In addition, metabolite variability arising from diurnal effects and diet were minimized, as the surgical procedures were performed in the morning on fasting patients.

During sample preparation for NMR, specimens were kept on ice to avoid changes in the metabolite levels due to enzyme activity. The pH of the samples was measured and was found to be within a narrow range (mean ± standard deviation SD, 7.4 ± 0.04). To avoid changes in the metabolite levels due to longer waiting time in the NMR autosampler, the number of samples chosen for each experimental procedure was at most 20 per experimental daily run. Also, all samples were kept at 4 °C in the autosampler before being recorded. Lastly, to avoid possible effects arising from variations in the NMR equipment, samples were randomly chosen for spectral acquisition.

**Fig. 2:**
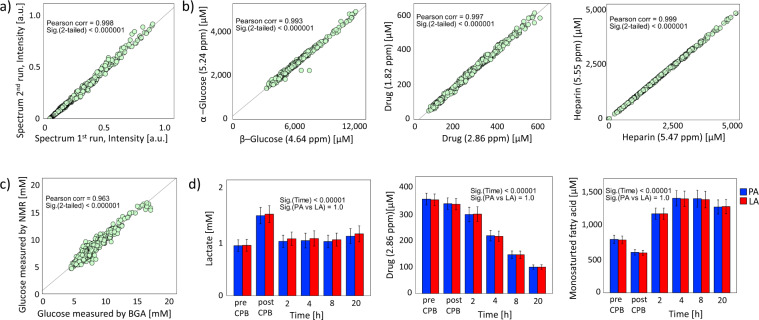
Data quality check. (**a**) For the within-day reproducibility, a blood sample collected from a patient was run twice, within an interval of 24 hours. High correlation is observed between two representative spectra of same patient (R^2^ = 0.9998), indicating good within-day reproducibility. Diagonal line indicates perfect correlation between samples. (**b**) The fitting accuracy of several metabolites was demonstrated by regressing two quantified peak areas of the same metabolite found at different regions on the NMR spectra. A good correlation was observed. (**c**) The quality of NMR measurements and of quantification accuracy was checked by comparing glucose concentration obtained from measuring thawed serum samples on the 600 MHz NMR with the full fresh arterial blood measured on the standard blood gas analyser (BGA) at the hospital. A high correlation is observed between instruments (Pearson correlation = 0.963, p < 0.00001). (**d**) Example of metabolite changes with time as a consequence of ischemia-reperfusion due to the use of CPB showing similarities between left atrial (LA) and pulmonary artery (PA) samples. Bars represent means, while error bars are 95% confidence intervals.

To assure data quality, we used different strategies. First, the within-day reproducibility of NMR analysis was assessed. A total of 19 serum samples were run twice within an interval of 24 hours. A Pearson correlation analysis was performed on spectra intensities of same sample and a mean ± SD correlation coefficient of 0.992 ± 0.018 was found, indicating good within-day reproducibility. A representative example can be seen in Fig. 2a. Second, to assess the quantification accuracy of the fitted signals within a compound, two different signals arising from the same metabolite placed at different positions in the spectrum were compared (Fig. 1d). High fitting accuracies were found in all tested metabolites (Fig. 2b, Pearson correlation coefficient ≥ 0.97 with p < 0.00001), indicating consistent quantification. The quality of NMR measurements and metabolite quantification were further assessed by comparing serum glucose concentrations obtained through NMR measurements with fresh full arterial blood glucose measured by the standard blood gas analyser at the hospital (ABL 800 flex, Radiometer, Denmark). A correlation of 0.96 was obtained between both techniques (Fig. 2c) indicating high data quality. Finally, to ensure reliable metabolite identification, whenever necessary, additional confirmation was performed by spiking standard pure compounds into blood samples.

To screen for possible outliers, a PCA was performed. No outliers were detected and the PCA scores plot revealed that the major variance in the data was related to ischemia-reperfusion and longitudinal serial changes occurring postoperatively as an attempt to regain metabolic balance. A PLS regression analysis was performed to link changes in the metabolome during surgery with the duration of ischemia-reperfusion^8^. To test which metabolites significantly changed as a consequence of CPB during and after surgery in both the left atrium and pulmonary artery samples, paired t-tests and analysis of variance (ANOVA) with Tukey’s post-hoc test for multiple testing were used. Examples of representative longitudinal metabolite changes are visualised in Fig. 2d while additional statistical results are detailed in our previous publications^8,9^.

## Data Availability

All the pre-processing steps were performed using the available software TopSpin 3.1 (Bruker BioSpin, Germany), AMIX v.3.9.10 (Bruker BioSpin, Germany), and PLS-Toolbox 6.5 (Eigenvector Research, Wenatchee, WA, USA).
